# Vaginal leiomyoma in a goat expressing the nuclear progesterone receptor (PGR): a case report

**DOI:** 10.1186/s12917-024-04035-6

**Published:** 2024-05-07

**Authors:** Lukas Trzebiatowski, Mariusz Pawel Kowalewski, Sarah Schmid, Kirstin Skaar, Jana Müller, Axel Wehrend

**Affiliations:** 1 Veterinary Clinic for Reproductive Medicine and Neonatology, Justus Liebig University, Giessen, Germany; 2 Institute of Veterinary Anatomy, Vetsuisse-Faculty, University of Zurich, Zurich, Switzerland; 3 Institute for Veterinary Pathology, Justus Liebig University, Giessen, Germany

**Keywords:** Goat, Vaginal discharge, Vaginal tumor, Sex steroid hormone, Reproductive disorder

## Abstract

**Background:**

The risk of developing tumorous diseases in the genital tract also increases with age in animals. One of the classified tumor types is genital leiomyoma. Presently, our understanding of the pathogenesis of this tumor in goats is, however, limited. This accounts also for the information regarding the presence of steroid hormone receptors and, thus, possible responsiveness to circulating steroids.

**Case presentation:**

This study describes the case of a vaginal tumor in a seven-year-old Anglo-Nubian goat. The goat was presented due to blood mixed vaginal discharge. Per vaginal examination a singular pedunculated mass in the dorsum of the vagina measuring approximately 3 cm x 4 cm x 4 cm was revealed. After administering epidural anesthesia, the mass was removed electrothermally. There were no postoperative complications. The histopathological examination identified the mass as a leiomyoma. The immunohistochemical examination revealed the presence of the nuclear progesterone receptor (PGR) in the tumor tissue. One year after the surgery, during the follow-up examination, the goat was in good overall health, and the owners had not observed any recurrence of vaginal discharge.

**Conclusions:**

When observing vaginal discharge in goats, it is important to consider the possibility of genital tract tumors. These tumors may express sex steroid receptors. In the future, it is worth considering the investigation of potential approaches for preventing tumorigenesis or treating the tumor, such as castration or the administration of antiprogestogens.

## Background

As goats age, the risk of developing neoplastic diseases increases. This is especially evident in the genital organs, where tumors of the cervix and uterus are frequently observed. Consequently, recently, some authors [1] have carried out reviews on the occurrence, locations, and types of genital tumors in goats. In this context, a frequently observed neoplasm is the leiomyoma, a benign tumor originating from smooth muscles, which has been well-documented in humans and various domestic mammals [2, 3]. Histologically, leiomyomas consist of smooth muscle cells as main cellular component and may be diagnosed as fibroleiomyoma if a significant amount of fibrous connective tissue is present.

However, the current understanding of the etiology behind the initiation and growth of genital tumors, including leiomyomas, in goats remains limited. Previous studies have indicated that genital tumors of female dogs and humans express sex steroid receptors [3] and might exhibit responses to sex steroids. It is assumed that progesterone stimulates tumor growth by regulating growth factor function, extracellular matrix activity, microRNA expression, and downregulating of tumor necrosis factor - α [4]. However, such knowledge is currently absent in the case of goats.

## Case presentation

A seven-year-old female Anglo-Nubian goat was presented due to recurrent episodes of blood mixed vaginal discharge for examination in the Veterinary Clinic for Reproductive Medicine and Neonatology in Giessen. The goat had delivered two kids 16 weeks prior, and the parturition occurred without any complications.

**Fig. 1:**
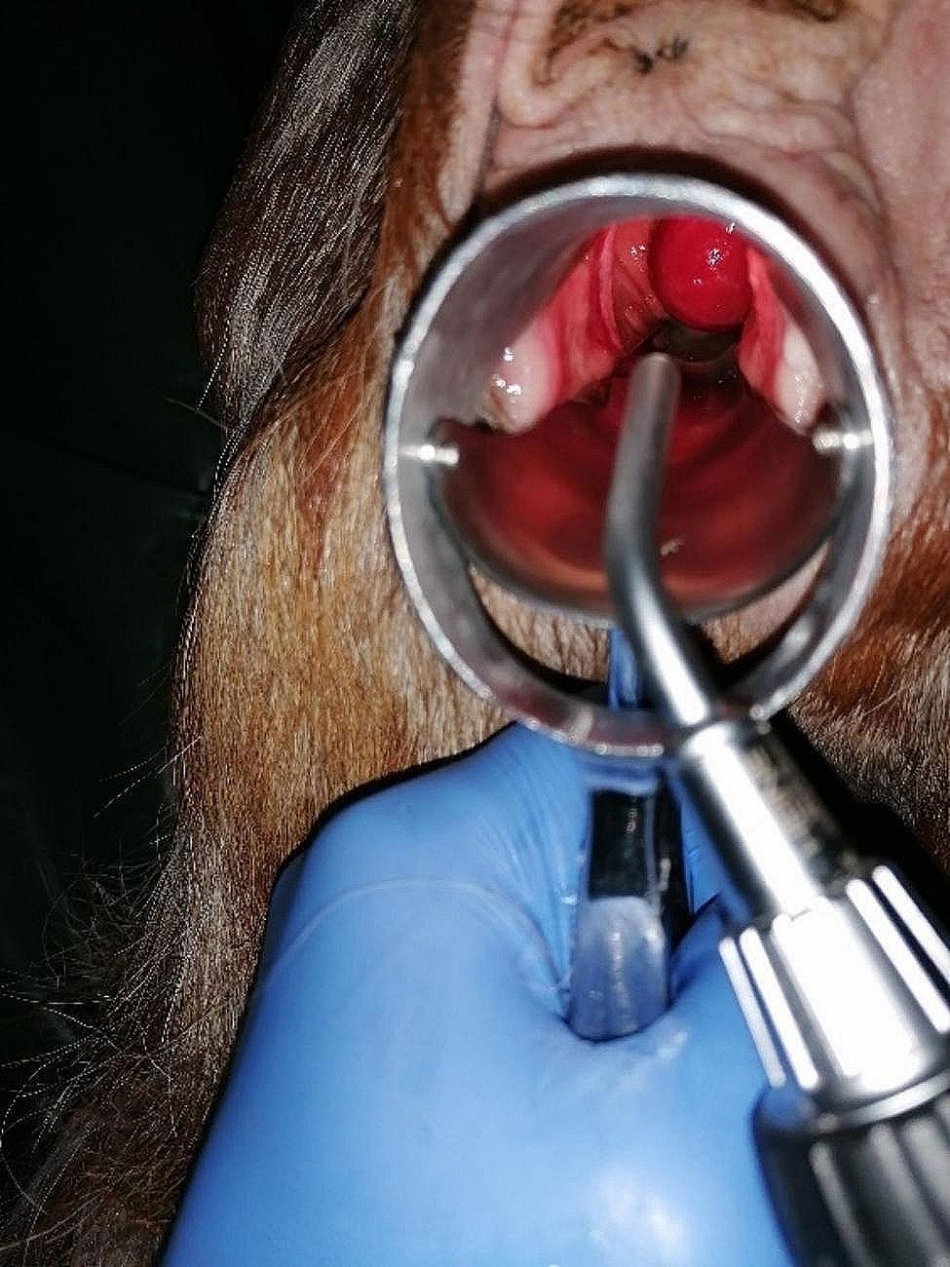
Visualization of the caudal part of the vaginal tumor through a stork’s beak speculum in a seven-year-old Anglo-Nubian goat

Upon presentation, the goat appeared to be in good general condition with vital signs in the normal range. Per vaginal examination vaginal discharge and a mass with superficial ulcerative changes measuring approximately 3 cm x 4 cm x 4 cm with a short pedicle protruding from the vaginal vault could be observed (Fig. 1). A transabdominal ultrasound of the abdomen and uterus was performed, but no specific findings were observed. Hematocrit and total protein levels were analyzed from the blood sample, showing a slightly decreased hematocrit (0.24 L/L) (reference range 0,28–0,40 L/L) and a total protein level within the reference (68 g/L) (reference range 64–70 g/L).

Prior to the surgery, the goat was administered 0.5 mg/kg of meloxicam (Melosolute, CP-Pharma, Burgdorf, Germany) and 30 mg/kg of metamizole (Metamizol WDT, WDT, Garbsen, Germany). Antibiotic coverage was provided with 7 mg/kg of amoxicillin (Betamox, Elanco Animal Health, Bad Homburg, Germany).

**Fig. 2:**
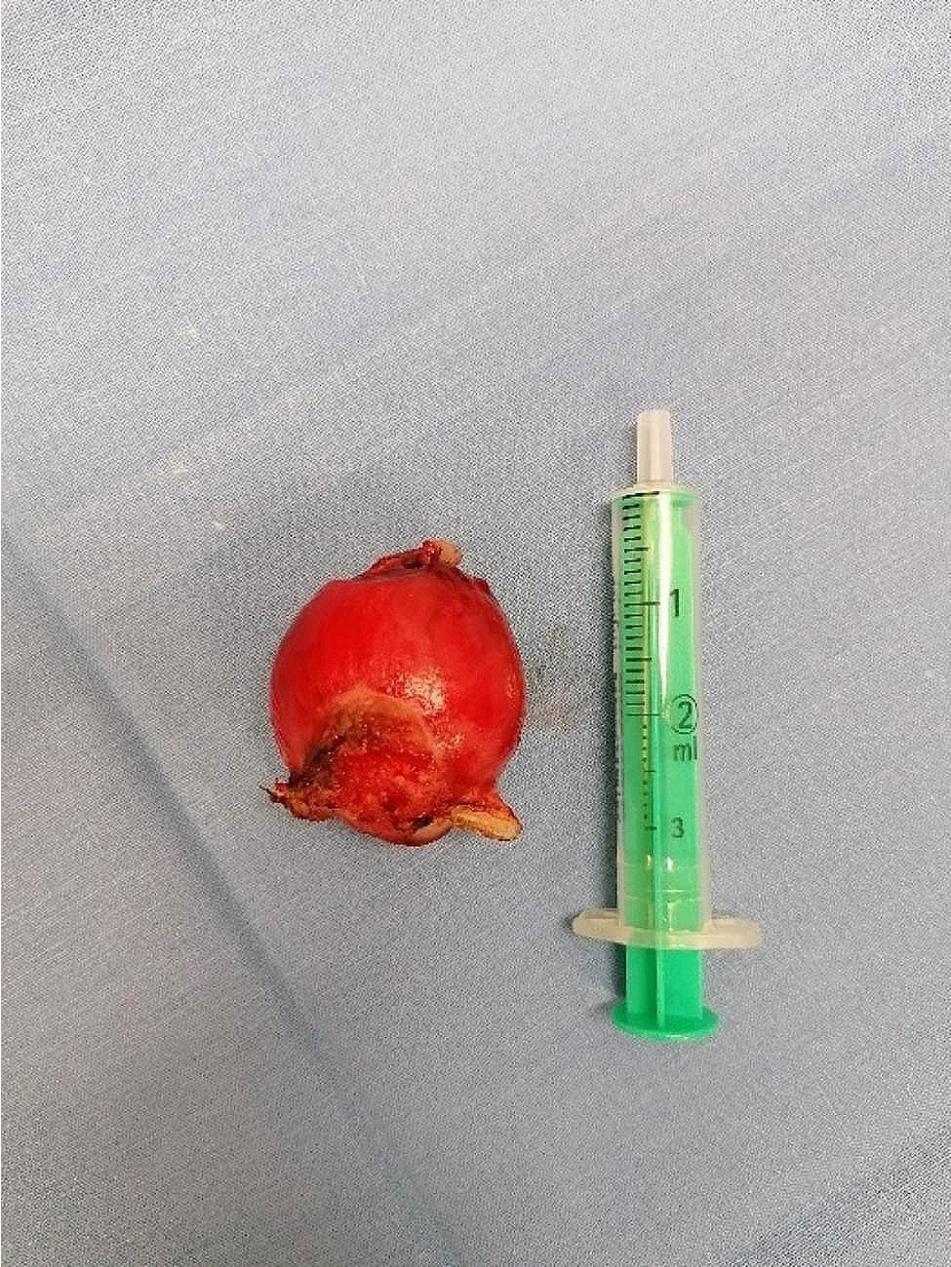
Vaginal tumor of a seven-year-old Anglo-Nubian goat after resection. At the bottom of the picture is the tumor`s coagulation site. The side to the top of the picture shows superficial ulceration

The tumor resection was performed under epidural anesthesia using 20 mg of procaine hydrochloride (Procamidor, WDT, Garbsen, Germany). A stork’s beak speculum was employed to spread the labia, and the tumor was drawn forward with an arterial clamp before electrothermally coagulating at the base using the bipolar tissue sealer/divider LigaSure LF1837^™^ (Medtronic, Meerbusch, Germany) with the energy platform ForceTriad^™^ (Medtronic, Meerbusch, Germany) and finally detached (Fig. 2). There was no postoperative bleeding, and the vaginal canal was flushed with 100 ml of sterile, isotonic sodium chloride solution (0.9% NaCl, B. Braun, Melsungen, Germany).

After the surgery, meloxicam treatment was continued for another day, and antibiotic administration was maintained for five days. On the third day, the surgical site was examined, and it showed signs of healing without any adverse reactions.

Five days after surgery, the goat was discharged in good general condition. A one-year follow-up was conducted, during which the goat remained out of breeding use, and no complications or recurrence of vaginal discharge were reported.

## Pathological findings

**Fig. 3:**
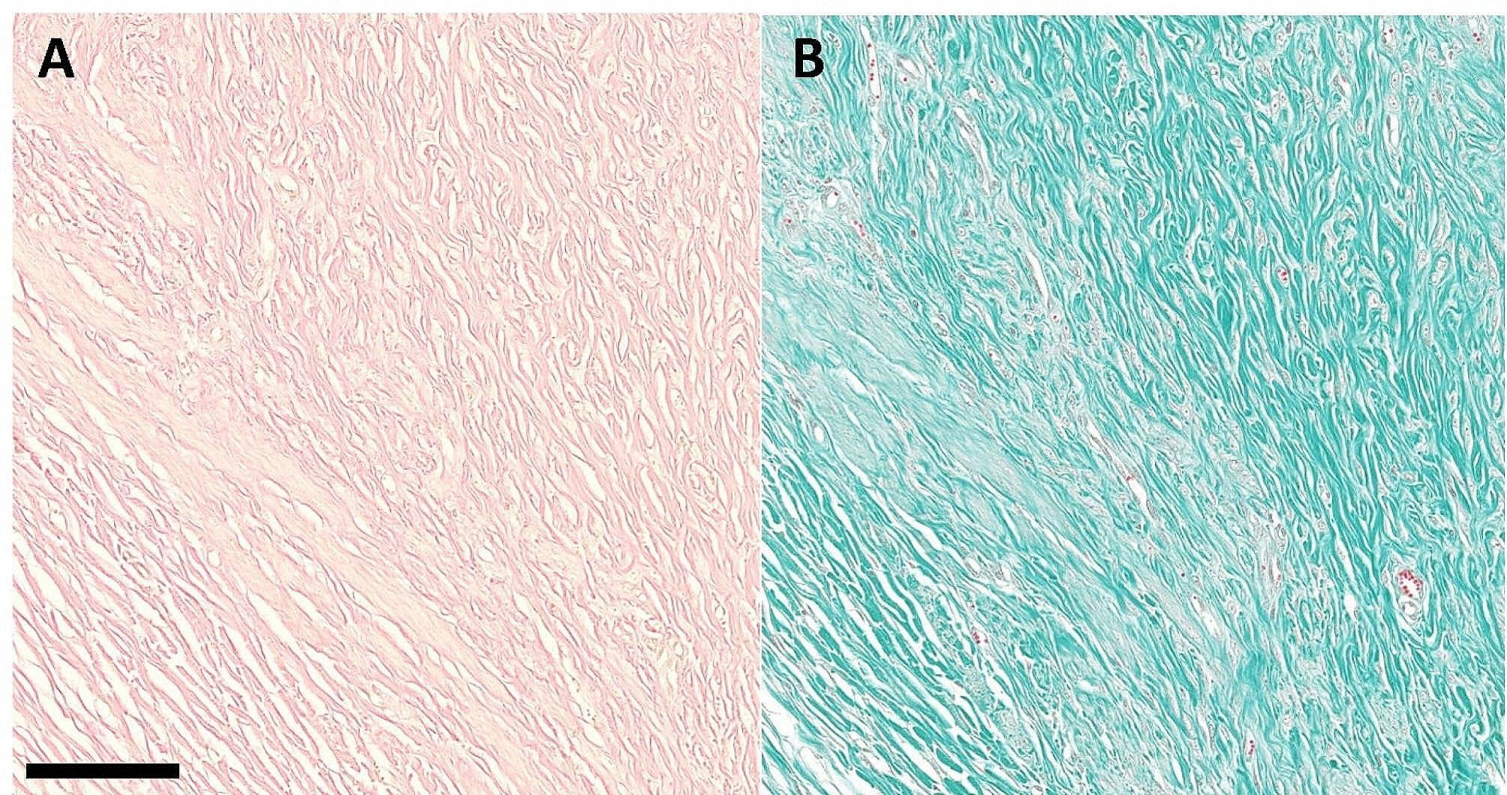
Specials stain of the leiomyoma: van Gieson’s **(A)** and Goldner stain **(B)**. Collagen fibers stain red and green, and cytoplasm of the neoplastic cells stains yellow and faint pink with van Gieson’s **(A)** and Goldner stain **(B)**, respectively. Bar = 100 μm

The vaginal mass was fixed in 10% neutral buffered formalin and submitted in total for histopathological examination. On gross examination, the mass was firm and inhomogeneously white on the cut section. Representative sections were routinely embedded in paraffin, sectioned 1 μm thick, stained with hematoxylin and eosin (HE), van Gieson’s and Goldner stain for evaluating the presence of smooth muscle cells and fibrous tissue. Histopathologically, there was a superficially ulcerated, unencapsulated, expansile, moderately cellular, well demarcated neoplasm composed of interlacing bundles of spindle-shaped cells on abundant fibrovascular stroma. Neoplastic cells had distinct cell borders, small to moderate amounts of eosinophilic cytoplasm, centrally located, elongated to cigar-shaped nuclei with vesicular chromatin and up to three small nucleoli. Anisokaryosis and anisocytosis were low, and mitoses were rare (< 1 in 2.37 mm²). The cytoplasm of the neoplastic cells stained yellow, and interspersed collagen fibers stained red with van Gieson’s and green with Goldner stain (Fig. 3), concluding the diagnosis of a leiomyoma.

## Immunohistochemical findings

The immunohistochemistry (IHC) of the neoplasm for the detection of nuclear progesterone receptors (PGR) was performed as described in [5], using an indirect immunoperoxidase detection method. The applied primary antibody was monoclonal mouse anti-human PGR IgG2a, IM1408-Clone PR10A9, IOPath®, Immunotech SAS, Marseille, France (dilution 1:300). The secondary antibody was biotinylated goat anti-mouse IgG, BA-9200-1.5, Vector Laboratories, Inc., Newark, USA. ImmPACT DAB Substrate kit, Peroxidase (HRP) (SK-4105, Vector Laboratories) was used for detection of signals. An isotype-specific irrelevant monoclonal antibody IgG2a Clone MOPPC-173 from EXBIO Praha, Vestec, Czech Republic was applied for negative control. The PGR antibody does not distinguish between the receptor isoforms PRA and PRB. The uterine tissue of a healthy goat was used as a positive control for PGR.

**Fig. 4:**
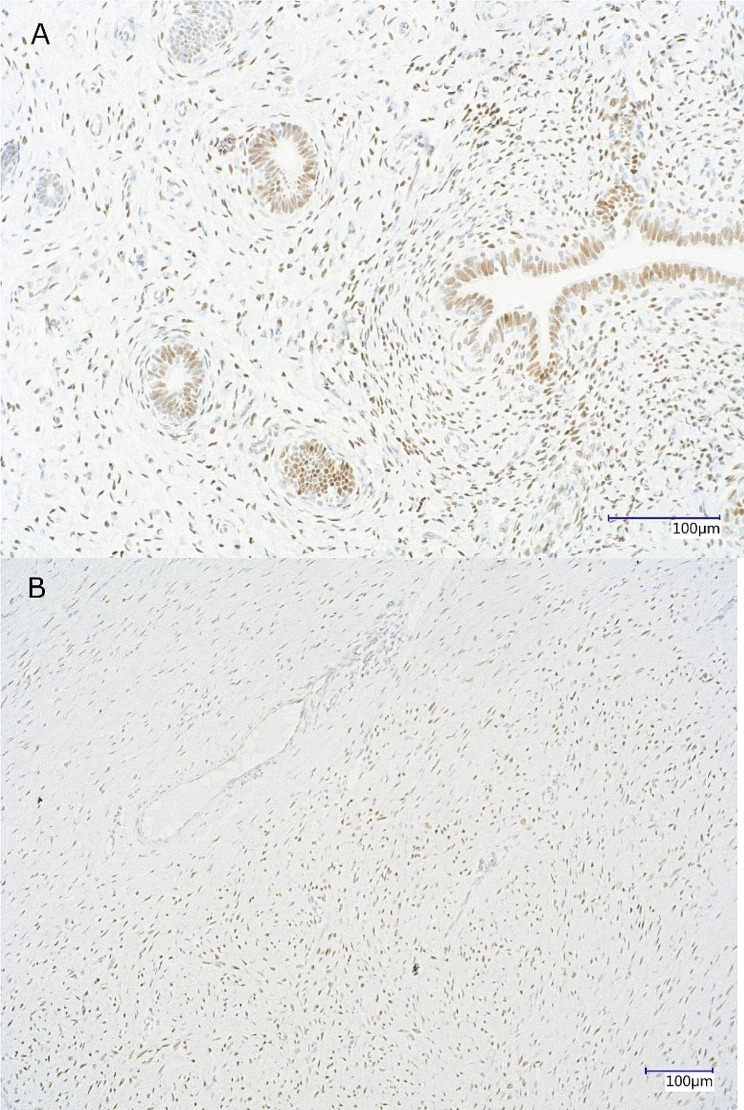
Immunohistochemical detection of PGR in the tumor (leiomyoma). A healthy caprine uterus was used as a positive control, showing a typical staining pattern of PGR **(A)**. The tumor shows distinct specific staining for the nuclear PGR **(B)**

As presented in Fig. 4, the tumor exhibited distinct positive staining for the nuclear PGR receptor, whereas negative control remained unstained. In positive controls specific stromal and epithelial endometrial staining was observed.

## Discussion and conclusions

In goats, pathological vaginal discharge can arise from abortions, postpartum retention, and other metropathies (e.g. mucometra) [6]. Additionally, tumors of the genital tract have been associated with vaginal discharge [1, 6]. In the case presented here, the blood mixed discharge was attributed to a vaginal tumor with superficial ulcerations.

The etiology of genital leiomyomas in humans has not been fully elucidated; however, influencing factors such as genetics and the presence of sex steroid receptors are known to play a role [4]. In goats, there have been no investigations into the underlying causes of these tumors. This case report represents the first evidence of the presence of nuclear progesterone receptors (PGR) in neoplastic smooth muscle cells of a vaginal leiomyoma in a goat.

Progesterone has been found to exert a proliferative effect on tumor cells in human uterine leiomyomas. In human medicine, the use of GnRH analogues to reduce hormone production in the ovaries has resulted in the regression of genital leiomyomas. Promising prospects lie in specific drug modulations of progesterone receptors [4].

In goats, the effectiveness of progesterone antagonists on progesterone receptors has been reported for inducing parturition, indicating that they could potentially be utilized for treating progesterone-responsive tumors.

Ovariohysterectomy could serve as a preventive measure or a valuable adjunct if a sex steroid-responsive tumor is suspected, especially if it is located at an inoperable site.

In the present case report, the tumor was resected due to its pedunculated nature, making it easily removable.

## Data Availability

All data generated or analyzed during this study are included in this published article.

## Abbreviations

PGR: Nuclear progesterone receptor
HE: Hematoxylin and eosin
IHC: Immunohistochemistry
